# An intervention for pulmonary rehabilitators to develop a social identity for patients attending exercise rehabilitation: a feasibility and pilot randomised control trial protocol

**DOI:** 10.1186/s40814-018-0238-z

**Published:** 2018-01-27

**Authors:** Andrew R. Levy, Bashir Matata, Sam Pilsworth, Adrian Mcgonigle, Lyndsey Wigelsworth, Linda Jones, Nicola Pott, Max Bettany, Adrian W. Midgley

**Affiliations:** 1 Department of Psychology, Edge Hill University, Ormskirk, L39 4QP UK; 2 Clinical Trials Unit, Liverpool Heart and Chest Hospital, Thomas Drive, Liverpool, L14 3PE UK; 3 Knowsley Community Respiratory Service, Liverpool Heart and Chest Hospital, Thomas Drive, Liverpool, L14 3PE UK; 4 Department of Sport and Physical Activity, Edge Hill University, Ormskirk, L39 4QP UK

**Keywords:** COPD, Group intervention, Identity, Quality of life

## Abstract

**Background:**

Chronic obstructive pulmonary disease (COPD) is a degenerative condition that can impair health-related quality of life (HRQoL). A number of self-management interventions, employing a variety of behavioural change techniques (BCTs), have been adopted to improve HRQoL for COPD patients. However, a lack of attention has been given to group management interventions with an emphasis on incorporating BCTs into rehabilitators’ practice. This study aims to pilot and feasibly explore a social identity group management intervention, delivered by COPD rehabilitation staff to patients attending exercise pulmonary rehabilitation. Doing so will help inform the plausibility of the intervention before conducting a full trial to evaluate its effectiveness to improve HRQoL.

**Methods:**

This is a two-centre, randomised cross-over controlled trial. Two pulmonary rehabilitation centres based in the UK will be randomly allocated to two treatment arms (standard care and intervention). Outcome measurements relating to HRQoL and social identity will be completed pre- and post-exercise rehabilitation. Focus group interviews will be conducted at the end of exercise rehabilitation to capture participants’ contextualised experiences of the intervention. COPD rehabilitators will undertake semi-structured interviews at the end of the trial to garner their holistic perspectives of intervention fidelity and implementation.

**Discussion:**

This is the first study to adopt a social identity approach to develop a rehabilitator-led, group management intervention for COPD patients attending exercise pulmonary rehabilitation. The results of this study will provide evidence for the feasibility and sample size requirements to inform a larger study, which can ascertain the intervention’s effectiveness for improving HRQoL for COPD patients.

**Trial registration:**

ClinicalTrials.gov NCT02288039. Date 31 October 2014

## Background

Chronic obstructive pulmonary disease (COPD) is a chronic debilitating condition, and it is estimated that by 2020, COPD will be the fifth most burdensome disease and the third leading cause of mortality worldwide [1]. In view of the fact that COPD is a progressive disability, many individuals will experience the slow development of functional impairment and subsequently a deterioration of health-related quality of life (HRQoL). Therefore, HRQoL in COPD sufferers has become an important treatment outcome [2].

Since there is currently no cure for COPD, self-management of the disease is essential for reducing symptoms and improving the HRQoL. The goal of self-management is for patients to acquire the skills needed to carry out disease-specific medical regimes, to guide changes in health behaviour and to provide emotional support to enable patients to adjust their roles for optimal function and control of their disease [3]. A plethora of self-management interventions to improve HRQoL among COPD patients has been developed and empirically tested. A meta-analysis of randomised controlled trials (RCTs) and non-RCTs revealed self-management-based interventions improve HRQoL in COPD populations over and above standard care provision [4]. More recently, Newham and colleagues [5] concluded self-management interventions can be more effective than usual care for improving HRQoL; however, their effectiveness was variable.

A number of recent reviews have attempted to explore the content underpinning self-management interventions [5, 6]. Notably, the content of self-management interventions has comprised of a number of behavioural change techniques such as patient education, action planning, goal setting and biofeedback. The delivery of these techniques was largely conducted by non-clinical staff through individual sessions either face to face or telephone conversations or through the individual distribution of booklet information. Based on the aforementioned reviews, two eminent issues arise. First, it is not surprising that self-management interventions have largely taken an individualistic approach given that sufferers of COPD are diagnosed and treated as individuals in hospital, or when visiting the doctor. Group contexts, however, such as exercise rehabilitation, can provide an additional opportunity to improve the HRQoL for COPD sufferers through the group management of COPD treatment. To date, the utilisation of group contexts to facilitate group management interventions has largely remained unexplored. Given that COPD sufferers can be stigmatised [7], they may consequently experience impaired social interactions and isolation [8]. Group management interventions, therefore, have the potential to promote positive social relationships and mutual social support for fellow COPD sufferers, providing an alternative contribution towards their HRQoL. Second, there is a notable lack of clinical staff involved in the delivery of group management interventions within the COPD literature. In the context of pulmonary exercise rehabilitation, COPD rehabilitators are uniquely positioned to make an important contribution to the delivery of behaviour change techniques given their active engagement in exercise rehabilitation. Nurse-led self-management interventions have been found to reduce symptoms and improve HRQoL among COPD patients [9]. However, to date, no research has explored the feasibility for involving COPD rehabilitators in the delivery of group management interventions in the context of pulmonary exercise rehabilitation.

A theoretical approach not yet considered for the group management of COPD is social identity theory [10]. The key premise of social identity theory is that group membership (e.g. exercise group) to which a person belongs can provide an individual with a sense of who they are in terms of a defined group identity (i.e. ‘we’ and ‘us’ rather than ‘I’ and ‘me’), that is, the way a person feels and thinks about self is derived from their social groupings. According to social identity theorising, a group identity is formed on the basis of three contextually salient social processes: (1) *categorisation*: awareness of similarities that connect group members as a team; (2) *identification*: positively valuing the importance of belonging to a group membership and (3) *inter-group comparison*: group membership perceived as more favourable in comparison with other out-groups. In group contexts, the social processes underpinning social identity theorising can serve to enhance group members’ self-esteem and sense of connectedness to others and provide a basis for receiving social support [11]. Furthermore, identifying with activities that are congruent to the group can facilitate adaptive cognitive, emotional and physical well-being within individuals as a result of favourable social group exchanges [11]. Therefore, the development of social identity within a group setting can be adaptive for one’s well-being and achieving desirable health outcomes.

In the context of health and well-being, there is emerging evidence for interventions that draw upon the social identity approach [12–16]. Collectively, these findings suggest that social identity has benefits for well-being among older adults, in part, because groups facilitate communication and engagement with information, alongside providing a basis for providing social support and positive social integration. In view that COPD mainly affects older adults, it is possible that group contexts that develop a social identity can make an important contribution to their HRQoL. Therefore, the purpose of the present study is to feasibly examine a pilot RCT rehabilitator-led social identity intervention, in order to obtain data for a future effectiveness trial to improve the HRQoL for COPD patients attending exercise pulmonary rehabilitation. Specifically, the feasibility and pilot RCT will determine:

1. The required sample size for a larger RCT trial to establish effectiveness
2. Recruitment, retention and adherence rates, alongside identifying the practicalities and strategies to facilitate these rates for COPD patients
3. The appropriateness of outcome measures and randomisation in terms of:

- Patient willingness to complete outcome measures (e.g. percentage of completed data and missing data)
- Rehabilitator willingness for collecting outcome data (e.g. percentage of completed and missing case report forms)
- Rehabilitator and patient willingness to be randomised across centres

4. The acceptability of the intervention for patients, with respect to:

- Engagement and compliance to the intervention protocol
- Perceptions of barriers and facilitators for COPD patients
- The degree of patient satisfaction
- The perceived relevance of the intervention

5. The acceptability for rehabilitators delivering the intervention, in terms of:

- Intervention fidelity (e.g. compliance with training protocol, monitoring of intervention delivery competency)
- Provision of resources for intervention implementation
- Perceptions of barriers and facilitators for practice change

## Methods

### Study design and setting

**Fig. 1:**
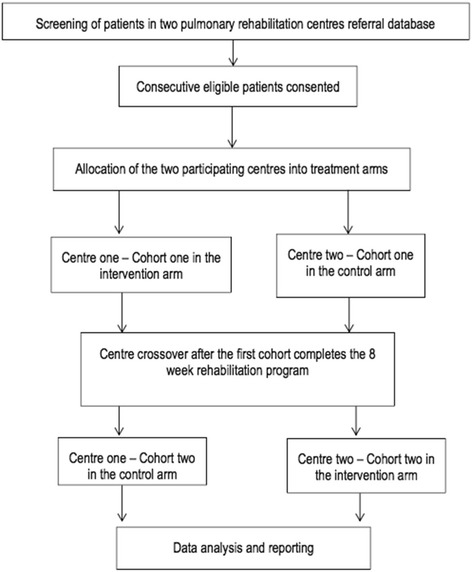
Study flow chart

We propose a pilot, two-centre, randomised cross-over controlled trial. Two pulmonary rehabilitation centres based in the UK will be randomly allocated to two treatment arms (centres are units of randomisation, not the patients). A total of 42 consecutive eligible participants undergoing an 8-week pulmonary exercise rehabilitation at two centres will be consented as the first trial cohort. The participants in one centre will be given standard care (*n* = 21), and the participants in the second centre will be given the intervention (*n* = 21). The standard care arm will consist of participants receiving their standard pulmonary exercise rehabilitation programme (the control group), and the intervention arm will consist of patients receiving the standard pulmonary rehabilitation programme plus a rehabilitator-led intervention aimed at developing a social identity (the intervention group). After the first cohort completes their 8-week rehabilitation programme, the centres will switch study arms and a second cohort of 42 new eligible participants will be recruited. This cross-over design aims to achieve symmetry of any confounders relating to rehabilitation centre location. Quantitative outcome measures will be determined at two time points—before rehabilitation (week 0) and at the last week of rehabilitation (week 8). Participants will also be invited to attend focus groups at the end of the rehabilitation period (week 9) to obtain a qualitative evaluation of the intervention. Obtaining both quantitative and qualitative information will help establish a clear and comprehensive plan for progression into a future full RCT trial (see Fig. 1 for an overview of the study design).

### Sample size estimation

As this is a feasibility and pilot study, in conjunction with adopting a novel intervention that has not been tested with COPD patients, no formal sample size calculation will be conducted. We aim to recruit 60 COPD patients within 6 months. This sample size is in accordance with recommendations for parameter estimation from pilot studies in order to determine an appropriate sample size for a full clinical trial [17]. However, in order to mitigate the known high dropout rate due to high incidences of acute exacerbations for COPD patients during pulmonary rehabilitation, an additional 24 patients (40%) will be recruited.

### Participant recruitment and eligibility

In this feasibility and pilot study, we plan to recruit 84 consecutive eligible COPD patients attending pulmonary exercise rehabilitation. Rehabilitators will recruit participants who have been referred to pulmonary exercise rehabilitation. Prior to embarking upon exercise pulmonary rehabilitation, rehabilitators will provide each participant with an information sheet providing full details of the study. Those participants willing to take part will be asked to complete a consent form. To be eligible to take part in this study, participants must (1) have been diagnosed as having COPD and enrolled on a pulmonary rehabilitation programme; (2) score between 1 and 4 on the modified medical research council dyspnoea scale; (3) have FEV_1_/FVC < 0.7 on spirometric recording, as per NICE guidance; and (4) willingly provide written informed consent.

### Description of the intervention

Based on a literature review and tenets of social identity theory, the intervention will train COPD rehabilitators to create a social identity for COPD patients attending exercise rehabilitation. Social identity will be operationalised based on the technique of group goal setting. There is an increasing body of research to support the theoretical integration of social identity principles and group goal setting [18, 19]. In the context of the current intervention, for example, group-based goals will serve to (1) increase the likelihood that COPD patients will define themselves as members of the same exercise rehabilitation group, ‘we’ or ‘us’ (i.e. categorisation); (2) provide a sense of common fate and collective group purpose and meaning (i.e. identification) and (3) define exercise rehabilitation as a distinct social in-group compared to other social out-groups (i.e. inter-group comparison).

COPD rehabilitators will be invited to attend two workshops, each lasting 2 h. The first workshop will focus on knowledge dissemination of social identity principles in the context of exercise pulmonary rehabilitation. Its objective will be to ensure COPD rehabilitators understand social identity and how its principles relate to COPD patients attending exercise rehabilitation. The second workshop will be concerned with the practical application of social identity through collaborative SMART group goal setting, in exercise pulmonary rehabilitation. Its objective will be for COPD rehabilitators to understand how to develop social identity using the technique of group goal setting for COPD patients during exercise rehabilitation. Both workshops will be classroom-based and will be delivered using a range of visual aids including PowerPoint slides and video clips. Group activities in the form of discussions, brainstorming, scenario problem solving and reflexivity will be incorporated into both workshops. Prior to each workshop, COPD rehabilitators will be provided with training packs that contain PowerPoint slides, workshop notes, planning sheets and guides for monitoring and evaluating goal progress. The workshop instructor will be a member of the research team who is a qualified practitioner psychologist with the Health and Care Professions Council in the UK. The instructor will be available to provide immediate feedback and answer any questions the COPD rehabilitators may have over the course of the workshops.

### Data collection

Baseline questionnaire data will be collected during participants’ first pre-rehabilitation assessment visit (week 0; time 1), including demographic data, anthropometrics, smoking status and medical history. Outcome measures recorded during the assessment visit will be the COPD Assessment Test (CAT), Chronic Respiratory Questionnaire-Self Report (CRQ-SR), Lung Information Needs Questionnaire (LINQ) and the modified Medical Research Council (mMRC) scale. Objective data will be collected via a participant six-minute walk test (6MWT). Outcome measures will be re-administered during a follow-up assessment, 1-week post-rehabilitation completion (week 9; time 2). Additionally, baseline data will be collected during the first rehabilitation session (week 1; time 1) that will include outcome measures pertaining to EuroQoL-5 Dimensions-5 Levels (EQ-5D-5L), Hospital Anxiety Depression Scale (HADS) and In-Group Identification Scale (IGIS) and re-administered during the last rehabilitation session (week 8; time 2). Focus group interviews with participants from the intervention group will be conducted during their follow-up assessment (week 9), and individual interviews with the rehabilitators will happen immediately at the end of the trial period.

#### Outcome measures

##### CAT

The CAT was designed to assess COPD-specific HRQoL and consists of eight items presented as a semantic 6-point differential scale. Items assess cough, production of phlegm, chest tightness, breathlessness, activity limitations, confidence, sleep and energy. The minimum score possible for each item is 0, and the maximum score is 5. As such, the overall score can range between 0 and 40. The CAT has demonstrated good reliability (Cronbach coefficient alpha = 0.88) and good test-retest reliability (intraclass correlation coefficient = 0.80) [20].

##### CRQ-SR

The CRQ-SR measures HRQoL in patients with COPD. It contains 20 items that assess dyspnoea (5 items), fatigue (4 items), emotional function (7 items) and mastery (4 items). Patients select, rank and score five everyday activities that make them breathless on a 7-point Likert scale from 1 (extremely short of breath) to 7 (not at all short of breath). Scores are calculated by dividing the total score per domain by the corresponding number of items. Therefore, overall scores can range, per domain, between 1 and 7 with lower scores indicating a greater degree of dysfunction. The CRQ-SR has demonstrated good reliability across all domains with Cronbach coefficient alphas ranging between 0.75 and 0.91 [21]. The CRQ-SR intraclass correlation coefficient ranges between 0.83 and 0.90 [22], demonstrating very good test-retest reliability.

##### LINQ

The LINQ is an information needs patient-centred instrument, assessing COPD disease-specific knowledge and management. It contains 16 items and is composed of six subscales including disease knowledge, medication, self-management, smoking, exercise and diet. Each item is multiple choice and scored where 0 = no information need and 1–3 = an informational need. Scores are summed for each subscale and can range from 0 to 4 (disease knowledge), 0–5 (medication), 0–6 (self-management), 0–3 (smoking), 0–5 (exercise) and 0–2 (diet). Higher scores reflect a greater informational need. The LINQ has been found to have good test-retest reliability (interclass correlation coefficient 0.66–0.98) and an overall total score Cronbach alpha coefficient of 0.62 [23].

##### mMRC

The mMRC is a measure of perceived respiratory disability for daily activities. It consists of five descriptive breathlessness statements graded 0 (not troubled by breathlessness) to grade 4 (being too breathless) and is used extensively as an evaluation rating for dyspnoea [24] and a valid tool to assess disability in patients with COPD [25]. Furthermore, Hsu and colleagues [26] have concluded that the mMRC is a concise and practical tool to assess the HRQoL of COPD patients in daily clinical practice.

##### EQ-5D-5L

The EQ-5D-5L is a generic measure of HRQoL. It consists of five dimensions, mobility, self-care, usual activities, pain/discomfort and anxiety/depression. Each dimension has a five-level response option ranging from 1 (no problems) to 5 (extreme problems). Scores across the five-level responses are combined to produce a five-digit number that is converted to a utility index based on the EQ-5D-5L value set for England [27]. The utility index ranges from − 0.208 (worst possible health) to 1.000 (best possible health). The EQ-5D-5L also includes a 20-cm visual analogue scale that records patient’s self-rated health with end points ranging from 0 (the worst health you can imagine) to 100 (the best health you can imagine). A recent study by Nolan et al. [28] validated the use of the EQ-5D-5L as a measure for HRQoL with COPD patients.

##### HADS

The HADS was developed to detect states of depression and anxiety among patients in clinical settings. It contains 14 items and consists of two subscales, anxiety and depression. The items are rated on a 4-point Likert scale ranging from 0 (not present) to 3 (considerable). Item scores are summed giving separate scores for anxiety and depression ranging from 0 to 21. The psychometric properties for the use of the HADS among COPD patients have previously been successfully established [29]. Previous research has indicated anxiety and depression, as measured by HADS, to be associated with HRQoL for respiratory conditions [30, 31].

##### IGIS

The IGIS is a 14-item multidimensional measure of social identity-related processes. Specifically, it measures social processes of identification towards a group membership across five domains, solidarity (3 items), satisfaction (4 items), centrality (3 items), individual stereotyping (2 items) and in-group homogeneity (2 items). In this study, group membership will be associated with the COPD exercise rehabilitation group and will be referred to as such in all items. Each item will be rated on a 7-point Likert scale anchored at 1 (strongly disagree) to 7 (strongly agree). Scores will be calculated by dividing the total score per domain by the corresponding number of items. Therefore, scores can range between 1 and 7 with higher scores indicating greater group identification. Leach and colleagues [32] have shown the IGIS to be reliable and valid.

##### 6MWT

The 6MWT is a functional exercise capacity test approved by the American Thoracic Society for use with COPD patients. The objective of this test is for patients to walk as far as possible during 6 min over a 30-m-marked stretch. Upon test completion, the total distance covered will be calculated. Previous research has identified that exercise capacity, as determined by the 6MWT, is an important physiological factor that can determine HRQoL [33].

#### Interviews

At the end of each 8-week exercise rehabilitation programme, the intervention group will undertake a focus group interview. The group interviews will primarily focus on the acceptability of the intervention from COPD patients’ perspectives. In addition, to ascertain COPD rehabilitator perspectives of intervention acceptability, individual semi-structured interviews will be conducted once the entire rehabilitation period has been completed.

### Data analyses

Prior to data analyses, quantitative data will be screened for data entry accuracy (approximately 10% of the sample), out of range values and missing data. The mean and standard deviation (median and interquartile range for non-normally distributed data) and percentages will be used to calculate participant recruitment, outcome measure completion, rates of retention and adherence and pre- and post-intervention values for all outcome values. Since this is a pilot study, treatment comparisons will focus on effect size estimation and associated 95% confidence intervals rather than hypothesis testing, as recommended [17]. A systematic inductive thematic approach [34] will be used to analyse patient focus group interviews and rehabilitator individual semi-structured interviews.

## Discussion

Involving COPD rehabilitators in the delivery of group management interventions can potentially offer an alternative way for improving HRQoL for COPD patients. This feasibility and pilot RCT will examine a social identity-derived group-based intervention, which is rehabilitator-led and delivered to COPD patients attending exercise pulmonary rehabilitation.

The proposed intervention is informed by a prominent theory (social identity) for understanding interpersonal relations. This is a key strength of the intervention as theoretical underpinning is essential for understanding the processes informing how and why the intervention may or may not work [35]. Furthermore, contextual factors may shape theoretical processes and therefore need to be fully understood to determine how interventions will work in real-world settings [35]. According to MRC guidance [35], interventions can be undermined by a lack of understanding concerning the context that interventions take place. As such, it is essential that the proposed intervention is developed with both theoretical and contextualised consideration in order to build an evidence base that informs COPD rehabilitator practice. The feasibility and pilot study proposed, therefore, is innovative as it will explore the compatibility of social identity theory in the context of pulmonary exercise rehabilitation for incorporating into rehabilitator-led practice. This information is paramount to determine if the proposed intervention can be feasibly done, and if so, how. As previously recommended [36], the pilot element will extend the feasibility component by conducting a small-scale study to determine randomisation and required sample size for a future larger scale RCT.

There has been an increasing number of attempts to develop social identity-based interventions [11]; however, such advances have not fed through to COPD rehabilitators working in pulmonary exercise rehabilitation. At present, there is no information available about social identity-derived behaviour change techniques to educate COPD rehabilitators. In order to bridge the gap between theory and practice, behaviour change techniques need to be feasibly developed and evaluated in order to empower COPD rehabilitators to operationalise social identity-based processes that can be implemented into group exercise rehabilitation. To this end, our protocol has described and rationalised the design of a study to pilot a new group-based intervention that integrates the social identity approach with group goal setting. The findings will inform a future RCT to test the effectiveness of a rehabilitator-led social identity-based intervention for improving the HRQoL of COPD patients attending exercise rehabilitation.

## Abbreviations

6MWT: Six-minute walk test
BCT: Behaviour change technique
CAT: COPD Assessment Test
COPD: Chronic obstructive pulmonary disease
CRQ-SR: Chronic Respiratory Questionnaire-Self Report
EQ-5D-5L: EuroQoL-5 Dimensions-5 Levels
HADS: Hospital Anxiety Depression Scale
HRQoL: Health-related quality of life
IGIS: In-Group Identification Scale
LINQ: Lung Information Needs Questionnaire
mMRC: modified Medical Research Council
MRC: Medical Research Council
RCT: Randomised control trial
SMART: Specific, measurable, attainable, realistic, timely

## References

[1] Vestbo J, Hurd SS, Agusti AG, Jones PW, Vogelmeier C, Anzueto A (2013). Global strategy for the diagnosis, management, and prevention of chronic obstructive pulmonary disease: GOLD executive summary. Am J Resp Crit Care.

[2] Weldam SW, Schuurmans MJ, Liu R, Lammers JW. Evaluation of quality of life instruments for use in COPD care and research: a systematic review. Int J Nurs Stud. 2013; 50: 688–707. https://doi.org/10.1016/j.ijnurstu.2012.07.017. 10.1016/j.ijnurstu.2012.07.017 22921317

[3] Effing TW, Bourbeau J, Vercoulen J, Apter AJ, Coultas D, Meek P (2012). Self-management programmes for COPD: moving forward. Chron Resp Dis.

[4] Zwerink M, Brusse-Keizer M, van der Valk PD, Zielhuis GA, Monninkhof EM, van der Palen J, et al. Self-management for patients with chronic obstructive pulmonary disease. Cochrane Libr. 2014;(Issue 3). 10.1002/14651858.CD002990.pub3 PMC7004246 24665053

[5] Newham JJ, Presseau J, Heslop-Marshall K, Russell S, Ogunbayo OJ, Netts P (2017). Features of self-management interventions for people with COPD associated with improved health-related quality of life and reduced emergency department visits: a systematic review and meta-analysis. Int J Chronic Obstr.

[6] Jonkman NH, Westland H, Trappenburg JC, Groenwold RH, Bischoff EW, Bourbeau J (2016). Characteristics of effective self-management interventions in patients with COPD: individual patient data meta-analysis. Eur Respir J.

[7] Lindqvist G, Hallberg LR (2010). ‘Feelings of guilt due to self-inflicted disease’ a grounded theory of suffering from chronic obstructive pulmonary disease (COPD). J Health Psychol.

[8] Johnson JL, Campbell AC, Bowers M, Nichol AM (2007). Understanding the social consequences of chronic obstructive pulmonary disease: the effects of stigma and gender. Proc Amer Thor Soc.

[9] Fletcher MJ, Dahl BH. Expanding nurse practice in COPD: is it key to providing high quality, effective and safe patient care? Prim Care Resp J. 2013; 22: 230–233. https://doi.org/10.4104/pcrj.2013.00044. 10.4104/pcrj.2013.00044 PMC6442791 23666716

[10] Tajfel H, Turner JC, Austin WG, Worchel S (1979). An integrative theory of intergroup conflict. The social psychology of intergroup relations.

[11] Haslam SA (2014). Making good theory practical: five lessons for an applied social identity approach to challenges of organizational, health, and clinical psychology. Brit J Soc Psychol.

[12] Gleibs IH, Haslam C, Jones JM, Haslam SA, McNeill J, Connolly H. No country for old men? The role of a ‘Gentlemen’s Club’ in promoting social engagement and psychological well-being in residential care. Aging Ment Health. 2011; 15: 456–66. https://doi.org/10.1080/13607863.2010.536137. 10.1080/13607863.2010.536137 21500012

[13] Gleibs IH, Haslam C, Haslam SA, Jones JM. Water clubs in residential care: is it the water or the club that enhances health and well-being?Psychol Health. 2011; 26:1361–1377. https://doi.org/10.1080/08870446.2010.529140. 10.1080/08870446.2010.529140 21598183

[14] Ysseldyk R, Haslam SA, Haslam C. Abide with me: religious group identification among older adults promotes health and well-being by maintaining multiple group memberships. Aging Ment Health. 2013; 17: 869–879. https://doi.org/10.1080/13607863.2013.799120. 10.1080/13607863.2013.799120 23711247

[15] Cruwys T, Haslam SA, Dingle GA, Jetten J, Hornsey MJ, Chonga EMD (2014). Feeling connected again: interventions that increase social identification reduce depression symptoms in community and clinical settings. J Affect Disorders..

[16] Haslam C, Cruwys T, Haslam SA, Dingle G, Chang MXL (2016). GROUPS 4 HEALTH: evidence that a social-identity intervention that builds and strengthens social group membership improves mental health. J Affect Disorders.

[17] Lancaster GA, Dodd S, Williamson PR (2004). Design and analysis of pilot studies: recommendations for good practice. J Eval Clin Pract.

[18] Haslam SA, Wegge J, Postmes T (2009). Are we on a learning curve or a treadmill? The benefits of participative group goal setting become apparent as tasks become increasingly challenging over time. Eur J Soc Psychol.

[19] Wegge J, Haslam SA, Haslam SA, van Kinppenberg D, Platow MJ, Ellemer N (2003). Group goal setting, social identity, and self-categorization. Social identity at work: developing theory for organizational practice.

[20] Jones PW, Harding G, Berry P, Wiklund I, Chen WH, Leidy NK (2009). Development and first validation of the COPD Assessment Test. Eur Respir J.

[21] Reda AA, Kotz D, Kocks JW, Wesseling G, van Schayck CP. Reliability and validity of the clinical COPD questionnaire and chronic respiratory questionnaire. Resp Med. 2010; 104: 1675–1682. https://doi.org/10.1016/j.rmed.2010.04.023. 10.1016/j.rmed.2010.04.023 20538445

[22] Williams JE, Singh SJ, Sewell L, Guyatt GH, Morgan MD. Development of a self-reported Chronic Respiratory Questionnaire (CRQ-SR). Thorax. 2001; 56: 954–959. https://doi.org/10.1136/thorax.56.12.954. 10.1136/thorax.56.12.954 PMC1745990 11713359

[23] Hyland ME, Jones RC, Hanney KE. The Lung Information Needs Questionnaire: development, preliminary validation and findings. Resp Med. 2006; 100: 1807–1816. https://doi.org/10.1016/j.rmed.2006.01.018. 10.1016/j.rmed.2006.01.018 16524709

[24] Mahler DA, Wells CK. Evaluation of clinical methods for rating dyspnea. Chest. 1988; 93: 580–586. https://doi.org/10.1378/chest.93.3.580. 10.1378/chest.93.3.580 3342669

[25] Bestall JC, Paul EA, Garrod R, Garnham R, Jones PW, Wedzicha JA. Usefulness of the Medical Research Council (MRC) dyspnoea scale as a measure of disability in patients with chronic obstructive pulmonary disease. Thorax 1999; 54: 581–586. https://doi.org/10.1136/thx.54.7.581. 10.1136/thx.54.7.581 PMC1745516 10377201

[26] Hsu KY, Lin JR, Lin MS, Chen W, Chen YJ, Yan YH (2013). The modified Medical Research Council dyspnoea scale is a good indicator of health-related quality of life in patients with chronic obstructive pulmonary disease. Singap Med J.

[27] Devlin N, Shah KK, Feng Y, Mulhern B, van Hout B. Valuing health-related quality of life: an EQ-5D-5L value set for England. In: In health economics and decision science discussion paper series. Sheffield: University of Sheffield; 2016. http://eprints.whiterose.ac.uk/97964/. 10.1002/hec.3564 PMC6680214 28833869

[28] Nolan CM, Longworth L, Lord J, Canavan JL, Jones SE, Kon SS (2016). The EQ-5D-5L health status questionnaire in COPD: validity, responsiveness and minimum important difference. Thorax.

[29] Bratås O, Grønning K, Forbord T (2014). Psychometric properties of the Hospital Anxiety and Depression Scale and The General Health Questionnaire-20 in COPD inpatients. Scand J Caring Sci.

[30] Cleland JA, Lee AJ, Hall S. Associations of depression and anxiety with gender, age, health-related quality of life and symptoms in primary care COPD patients. Fam Pract. 2007; 24: 217–223. https://doi.org/10.1093/fampra/cmm009. 10.1093/fampra/cmm009 17504776

[31] Kullowatz A, Kanniess F, Dahme B, Magnussen H, Ritz T. Association of depression and anxiety with health care use and quality of life in asthma patients. Resp Med. 2007; 101: 638–644. https://doi.org/10.1016/j.rmed.2006.06.002. 10.1016/j.rmed.2006.06.002 16891108

[32] Leach CW, Van Zomeren M, Zebel S, Vliek ML, Pennekamp SF, Doosje B (2008). Group-level self-definition and self-investment: a hierarchical (multicomponent) model of in-group identification. J Pers Soc Psychol.

[33] Moy ML, Reilly JJ, Ries AL, Mosenifar Z, Kaplan RM, Lew R (2009). Multivariate models of determinants of health-related quality of life in severe chronic obstructive pulmonary disease. J Rehabil Res Dev.

[34] Braun V, Clarke V. Using thematic analysis in psychology. Qual Res Psychol. 2006; 3: 77–101. https://doi.org/10.1191/1478088706qp063oa.

[35] Craig P, Dieppe P, Macintyre S, Michie S, Nazareth I, Petticrew M (2008). Developing and evaluating complex interventions: the new Medical Research Council guidance. BMJ.

[36] Eldridge SM, Lancaster GA, Campbell MJ, Thabane L, Hopewell S, Coleman CL, et al. Defining feasibility and pilot studies in preparation for randomised controlled trials: development of a conceptual framework. PLoS One. 2016;11: e0150205. https://doi.org/10.1371/journal.pone.0150205. 10.1371/journal.pone.0150205 PMC4792418 26978655

